# Rates of employment after liver transplant: A nationwide cohort study

**DOI:** 10.1097/HC9.0000000000000061

**Published:** 2023-02-20

**Authors:** Jacqueline B. Henson, Melanie Cabezas, Lisa M. McElroy, Andrew J. Muir

**Affiliations:** 1 Division of Gastroenterology, Department of Medicine, Duke University School of Medicine, Durham, North Carolina, USA; 2 Division of Gastroenterology, Department of Medicine, University of South Florida Health, Tampa, Florida, USA; 3 Division of Abdominal Transplant, Department of Surgery, Duke University School of Medicine, Durham, North Carolina, USA; 4 Duke Clinical Research Institute, Durham, North Carolina, USA

## Abstract

Employment outcomes after liver transplant (LT) over the past decade have not been described.

**Methods:**

LT recipients ages 18–65 from 2010-2018 were identified in Organ Procurement and Transplantation Network data. Employment within two years post-transplant was assessed.

**Results:**

Of 35,340 LT recipients, 34.2% were employed post-LT, including 70.4% who were working pre-transplant, compared to only 18.2% not working preLT. Younger age, male sex, educational attainment, and functional status were associated with returning to employment.

**Conclusion:**

Returning to employment is an important goal for many LT candidates and recipients, and these findings can be used to guide their expectations.

## INTRODUCTION

Returning to work after liver transplantation (LT) is a common goal of transplant candidates.^1^ Yet, fewer than one-third of the recipients are employed posttransplant, and this is associated with dissatisfaction in the LT recipients.^1^–^3^ While a few studies of post-LT employment have been performed, many of these have been single-center, and the most recent analyses of the US national data included employment outcomes only as of 2009.^2^,^3^ Importantly, the LT landscape has changed over the past decade, and a more contemporary assessment of post-LT employment is thus needed to appropriately counsel the transplant candidates and recipients.

## METHODS

We performed this study using data from the Organ Procurement and Transplantation Network after 2009 to characterize employment post-LT and to identify factors associated with posttransplant employment. We identified first-time, non-status 1, single organ LT recipients of ages 18–65 from 2010 to 2018 with at least 6 months of follow-up. Demographic and clinical variables were assessed at the time of transplant, and employment information was evaluated at the time of listing, transplant, and posttransplant. Working pre-LT was defined as employment at the time of listing or transplant. The primary outcome was employment posttransplant, which was defined as the employment within 2 years post-LT and was evaluated separately for individuals working and not working before transplant. Impaired functional status was defined as needing any assistance or a Karnofsky performance status <80%. Two time periods were defined, the first from 2010 to 2014 and the second from 2015 to 2018, each containing ~half of the total LTs in the cohort.

Demographic and clinical characteristics of LT recipients were compared by pretransplant and posttransplant employment status using Chi-square tests for categorical variables and Wilcoxon rank sum tests for continuous variables. Multivariable logistic regression was performed to identify predictors of an individual returning to employment posttransplant. Peri-transplant demographic and clinical characteristics were evaluated in univariable models and the predictors with *p*<0.10 were entered in the multivariable models and retained at a *p*<0.05 level of significance using a backward selection process. The time period was included in all models. This study was exempted from the institutional review board review.

## RESULTS

**FIGURE 1:**
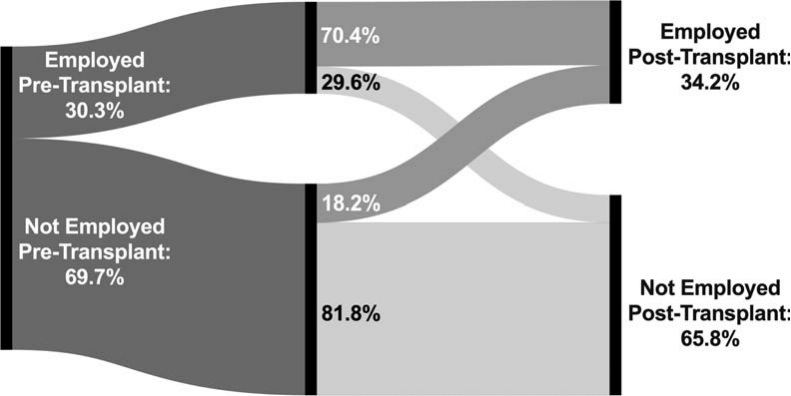
Posttransplant employment by pretransplant employment status.

A total of 35,340 LT recipients were included; 3023 were excluded due to missing pre-LT and post-LT employment information (Supplemental Figure; Supplemental Table 1, http://links.lww.com/HC9/A160). Overall, 34.2% of recipients were employed post-LT, and the majority had been employed pretransplant (62.7%). Among individuals working before transplant, 70.4% were working posttransplant, while only 18.2% of the individuals were not working in pre-LT achieved employment (Figure 1; Supplemental Table 2, http://links.lww.com/HC9/A160). Recipients who returned to employment were more likely to be younger, male, with greater educational attainment, and privately insured, and were less likely to be Hispanic, with diabetes and impaired functional status and in the intensive care unit (ICU) at transplant (Table 1). They also had lower Model for End-Stage Liver Disease (MELD) scores compared with those who did not return to work. Nearly 80% of the recipients aged younger than 50 or with postgraduate education regained employment post-LT. Individuals who were critically ill at transplant (on a ventilator or in the ICU) were the least likely to return to work, though the majority still had employment within 2 years. Recipients with autoimmune/cholestatic liver disease were more likely to return to employment, but the other etiologies, including alcohol-associated liver disease, were more similar.

**TABLE 1:** Factors associated with returning to work posttransplant

	Employed pre-transplant and posttransplant, n=7545, n (%)	Employed pre-transplant and not employed posttransplant, n=3173, n (%)	*P*	Employed pre-transplant and posttransplant, UV OR (95% CI)	*P*	Employed pre-transplant and posttransplant, MV OR (95% CI)
Age	—	—	<0.001	—	<0.001	—
18-39	856 (11.3)	216 (6.8)	—	Reference	—	Reference
40-49	1345 (17.8)	365 (11.5)	—	0.93 (0.77–1.12)	—	0.99 (0.81–1.21)
50-59	3400 (45.1)	1446 (45.6)	—	0.59 (0.50–0.70)	—	0.63 (0.52–0.75)
≥60	1944 (25.8)	1146 (36.1)	—	0.43 (0.36–0.50)	—	0.44 (0.36–0.53)
Sex	—	—	<0.001	—	<0.001	—
Male	5772 (76.5)	2183 (68.8)	—	1.48 (1.35–1.62)	—	1.58 (1.43–1.76)
Female	1773 (23.5)	990 (31.2)	—	Reference	—	Reference
BMI	—	—	0.84	—	0.84	—
Underweight	113 (1.5)	55 (1.7)	—	0.87 (0.62–1.21)	—	—
Normal	2038 (27.0)	859 (27.1)	—	Reference	—	—
Overweight	2666 (35.3)	1120 (35.3)	—	1.00 (0.90–1.12)	—	—
Obese	2728 (36.2)	1139 (35.9)	—	1.01 (0.91–1.12)	—	—
Ethnicity/race	—	—	<0.001	—	<0.001	—
Non-Hispanic White	5728 (75.9)	2259 (71.2)	—	Ref	—	Ref
Non-Hispanic Black	610 (8.1)	275 (8.7)	—	0.88 (0.75–1.02)	—	0.93 (0.79–1.10)
Hispanic	731 (9.7)	429 (13.5)	—	0.67 (0.59–0.76)	—	0.79 (0.69–0.92)
Other	476 (6.3)	210 (6.6)	—	0.89 (0.76–1.06)	—	0.82 (0.68–0.99)
Educational attainment^a^	—	—	<0.001	—	<0.001	—
Less than high school	140 (1.9)	117 (3.8)	—	0.60 (0.47–0.78)	—	0.78 (0.59–1.03)
High school or equivalent	2341 (32.0)	1182 (38.8)	—	Reference	—	Reference
College	3902 (53.4)	1480 (48.5)	—	1.33 (1.21–1.46)	—	1.22 (1.10–1.34)
Post-graduate	929 (12.7)	270 (8.9)	—	1.74 (1.49–2.02)		1.52 (1.29–1.78)
Payment source	—	—	—	—	<0.001	—
Private insurance	6791 (90.0)	2378 (74.9)	—	Reference	—	Reference
Public insurance	730 (9.7)	771 (24.3)	—	0.33 (0.30–0.37)	—	0.36 (0.32–0.41)
Other	24 (0.3)	24 (0.8)	—	0.35 (0.20–0.62)	—	0.36 (0.19–0.63)
Medical Condition at Transplant						
Diabetes^b^	—	—	<0.001	—	<0.001	—
Yes	1554 (20.6)	833 (26.3)	—	0.73 (0.66–0.80)	—	0.84 (0.75–0.94)
No	5981 (79.4)	2332 (73.7)	—	Reference	—	Reference
Prior ascites	—	—	<0.001	—	<0.001	—
Yes	4655 (61.7)	2126 (67.0)	—	0.79 (0.73–0.87)	—	—
No	2890 (38.3)	1047 (33.0)	—	Reference	—	—
Prior encephalopathy	—	—	<0.001	—	<0.001	—
Yes	3449 (45.7)	1681 (53.0)	—	0.75 (0.69–0.81)	—	—
No	4096 (54.3)	1492 (47.0)	—	Reference	—	—
Impaired functional status^c^	—	—	<0.001	—	<0.001	—
Yes	4473 (59.8)	2347 (74.9)	—	0.50 (0.45–0.55)	—	0.54 (0.48–0.60)
No	3003 (40.2)	786 (25.1)	—	Reference	—	Reference
Primary etiology of liver disease	—	—	<0.001	—	<0.001	—
Viral	1726 (22.9)	744 (23.4)	—	Reference	—	Reference
Alcohol-related	1132 (15.0)	517 (16.3)	—	0.94 (0.82–1.08)	—	0.94 (0.81–1.10)
NASH	741 (9.8)	362 (11.4)	—	0.88 (0.76–1.03)	—	0.95 (0.80–1.12)
Autoimmune/cholestatic	1345 (17.8)	418 (13.2)	—	1.39 (1.21–1.59)	—	1.21 (1.03–1.43)
HCC	1715 (22.7)	812 (25.6)	—	0.91 (0.81–1.03)	—	0.86 (0.75–0.98)
Other	886 (11.7)	320 (10.1)	—	1.19 (1.02–1.39)	—	1.05 (0.88–1.25)
On ventilator at transplant	—	—	<0.001	—	<0.001	—
Yes	90 (1.2)	82 (2.6)	—	0.46 (0.34–0.62)	—	—
No	7455 (98.8)	3091 (97.4)	—	Reference	—	—
In ICU at transplant	—	—	<0.001	—	<0.001	—
Yes	369 (4.9)	284 (9.0)	—	0.52 (0.44–0.61)	—	0.67 (0.55–0.81)
No	7176 (95.1)	2889 (91.0)	—	Reference	—	Reference
On dialysis at transplant^d^	—	—	<0.001	—	<0.001	—
Yes	321 (4.3)	235 (7.4)	—	0.56 (0.47–0.66)	—	—
No	7223 (95.7)	2938 (92.6)	—	Reference	—	—
MELD score at transplant median (IQR)	16 (10-24)	17 (11-25)	<0.001	0.99 (0.98–0.99)	<0.001	0.99 (0.99–1.00)
Transplant characteristics						
Donor type	—	—	0.008	—	0.01	—
Living donor	528 (7.0)	178 (5.6)	—	1.27 (1.06–1.51)	—	—
Deceased donor	7017 (93.0)	2995 (94.4)	—	Reference	—	—
Time period	—	—	0.44	—	0.44	—
2010-2014	3712 (49.2)	1535 (48.4)	—	Reference	—	Reference
2015-2018	3833 (50.8)	1638 (51.6 )	—	0.97 (0.89–1.05)	—	1.11 (1.01–1.22)

Note: Shown is a comparison of the characteristics of transplant recipients employed pretransplant by posttransplant employment status and a logistic regression model evaluating the factors associated with post-transplant employment in this population.

^a^ Missing n=357.

^b^ Missing n=18.

^c^ Missing n=109.

^d^ Missing n=1.

Abbreviations: BMI indicates body mass index; ICU, intensive care unit; IQR, interquartile range; MELD, Model for End-Stage Liver Disease score; MV, multivariable; UV, univariable.

## DISCUSSION

Our study shows that post-LT employment in this contemporary cohort has improved overall compared with the previously reported 24% from 2002 to 2008.^1^,^2^ The vast majority of recipients working posttransplant were employed at listing or at transplant. Among these individuals, the posttransplant employment rate was even higher at 70% and approached 80% in some groups. Recipients employed pre-LT and wishing to return to work posttransplant should therefore find these data encouraging. In contrast, becoming newly employed after LT was uncommon, occurring in only 18% of the recipients. Future research is needed to evaluate the barriers to employment in these patients, and the candidates not working before LT and desiring the posttransplant employment should be counseled on these potential difficulties.

The factors associated with returning to employment were overall similar to prior studies and included younger age, male sex, and greater educational attainment.^2^,^3^ In contrast to some previous studies, though, alcohol-associated liver disease was not associated with a decreased likelihood of post-LT employment.^2^ We also found that the degree of illness at transplant was associated with the likelihood of post-LT employment. Yet, an even stronger predictor was functional status: an impaired functional status was associated with 50% lower odds of returning to work post-LT. Functional status and the related concept of frailty have gained increasing attention as important determinants of both waitlist and posttransplant outcomes.^4^ These recipients likely had more difficulty recovering posttransplant and gaining sufficient fitness for work. Future studies should evaluate the effect of prehabilitation in more frail individuals on their ability to return to work. Recipients with impaired functional status hoping to return to work posttransplant, however, should be advised that they are likely to face challenges.

While the use of the Organ Procurement and Transplantation Network data allowed for an updated national assessment of posttransplant employment over the last decade, there are also inherent limitations associated with a retrospective review of the administrative databases, including missing, incomplete, or potentially inaccurate data. Employment data was not available on all transplant recipients, and we were also limited by the lack of additional information, such as whether post-LT employment was desired, the barriers when it was not achieved, and other factors that may be relevant that should be evaluated in future studies. Local job conditions are also likely important, and the changes in the job landscape as a result of the COVID-19 pandemic and the rise in remote work may impact the transplant recipients as well and will be important to assess in the future.^3^

In conclusion, in a contemporary transplant population, overall employment post-LT has improved, and 70% of the recipients with employment pretransplant returned to work within 2 years. New employment in the recipients not working before transplant is much less common, however. Both sociodemographic and clinical characteristics are associated with regaining employment after LT. Returning to work is an important goal for many LT candidates and recipients, and these findings can be used to guide their expectations.

## Supplementary Material
