# Supplementary material for: Rates of employment after liver transplant: A nationwide cohort study
Source: Hepatol Commun. 2023 Feb 20;7(3):e0061. doi: 10.1097/HC9.0000000000000061 (PMC9949776; doi:10.1097/HC9.0000000000000061)
Supplement: Supplementary file 1 [file hc9-7-e0061-s001.docx]

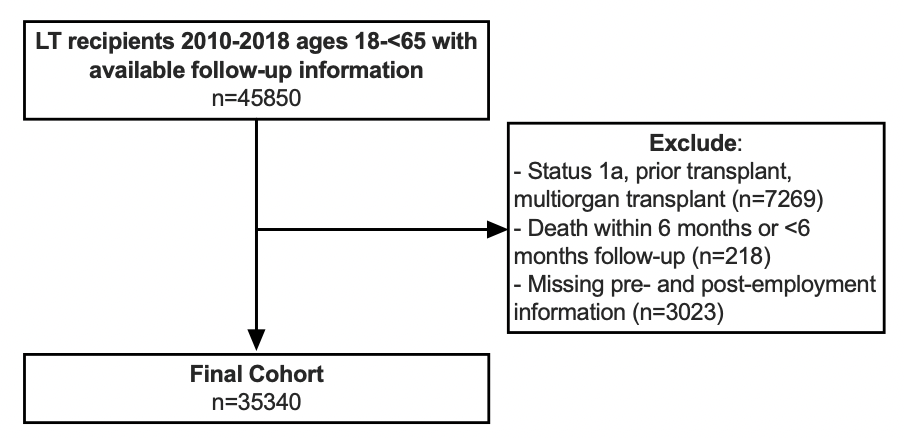


**Supplemental Figure 1.** Recipients included in study cohort. Abbreviations: LT, liver transplant.

Supplemental Table 1. Characteristics at Transplant by Pre-Transplant Employment Status.

|  | **All,**  n=35340, % (n) | **Employed pre-transplant,**  n=10718, % (n) | **Not employed pre-transplant,**  n=24622, % (n) | **p-value** |
| --- | --- | --- | --- | --- |
| Age |  |  |  | <0.001 |
| 18-39 | 9.0 (3190) | 10.0 (1072) | 8.6 (2118) |  |
| 40-49 | 15.5 (5470) | 16.0 (1710) | 15.3 (3760) |  |
| 50-59 | 44.3 (15669) | 45.2 (4846) | 44.0 (10823) |  |
| ≥60 | 31.2 (11041) | 28.8 (3090) | 32.2 (7921) |  |
| Sex |  |  |  | <0.001 |
| Female | 32.3 (11416) | 25.8 (2763) | 33.2 (8653) |  |
| Male | 67.7 (23924) | 74.2 (7955) | 66.8 (15969) |  |
| BMI |  |  |  | <0.001 |
| Underweight | 1.9 (669) | 1.6 (168) | 2.0 (501) |  |
| Normal | 26.1 (9225) | 27.0 (2897) | 25.7 (6328) |  |
| Overweight | 34.1 (12060) | 35.3 (3786) | 33.6 (8274) |  |
| Obese | 37.9 (13386) | 36.1 (3867) | 38.7 (9519) |  |
| Race/ethnicity |  |  |  | <0.001 |
| Non-Hispanic White | 71.5 (25255) | 74.5 (7987) | 70.1 (17268) |  |
| Non-Hispanic Black | 8.5 (3008) | 8.3 (885) | 8.6 (2123) |  |
| Hispanic | 14.4 (5108) | 10.8 (1160) | 16.0 (3948) |  |
| Other | 5.6 (1969) | 6.4 (686) | 5.2 (1283) |  |
| Educational attainment^a^ |  |  |  | <0.001 |
| Less than high school | 5.2 (1767) | 2.5 (257) | 6.4 (1510) |  |
| High school or equivalent | 43.6 (14730) | 34.0 (3523) | 47.9 (11207) |  |
| College | 44.5 (15014) | 51.9 (5382) | 41.1 (9632) |  |
| Post-graduate | 6.7 (2260) | 11.6 (1199) | 4.5 (1061) |  |
| Payment Source |  |  |  | <0.001 |
| Private insurance | 59.6 (21044) | 85.6 (9169) | 48.2 (11875) |  |
| Public insurance | 39.6 (14012) | 14.0 (1501) | 50.8 (12511) |  |
| Other | 0.8 (284) | 0.4 (48) | 1.0 (236) |  |
| **Medical Condition at Transplant** |  |  |  |  |
| Diabetes^b^ |  |  |  | <0.001 |
| Yes | 25.0 (8816) | 22.3 (2387) | 26.2 (6429) |  |
| No | 75.0 (26438) | 77.7 (8313) | 73.8 (18125) |  |
| Prior ascites |  |  |  | <0.001 |
| Yes | 74.0 (26155) | 63.3 (6781) | 78.7 (19374) |  |
| No | 26.0 (9185) | 36.7 (3937) | 21.3 (5248) |  |
| Prior encephalopathy |  |  |  | <0.001 |
| Yes | 61.3 (21658) | 47.9 (5130) | 67.1 (16528) |  |
| No | 38.7 (13682) | 52.1 (5588) | 32.9 (8094) |  |
| Impaired functional status^c^ |  |  |  | <0.001 |
| Yes | 81.0 (28416) | 64.3 (6820) | 88.3 (21596) |  |
| No | 19.0 (6662) | 35.7 (3789) | 11.7 (2873) |  |
| Primary etiology of liver disease |  |  |  | <0.001 |
| Viral | 25.1 (8872) | 23.0 (2470) | 26.0 (6402) |  |
| Alcohol-related | 23.6 (8354) | 15.4 (1649) | 27.2 (6705) |  |
| NASH | 11.3 (3988) | 10.3 (1103) | 11.7 (2885) |  |
| Autoimmune/cholestatic | 11.5 (4063) | 16.4 (1763) | 9.3 (2300) |  |
| HCC | 18.5 (6552) | 23.6 (2527) | 16.2 (3995) |  |
| Other | 10.0 (3541) | 11.2 (1206) | 9.5 (2335) |  |
| On ventilator |  |  |  | <0.001 |
| Yes | 3.1 (1108) | 1.6 (172) | 3.8 (936) |  |
| No | 96.9 (34232) | 98.4 (10546) | 96.2 (23686) |  |
| In ICU at transplant |  |  |  | <0.001 |
| Yes | 10.8 (3823) | 6.1 (653) | 12.9 (3170) |  |
| No | 89.2 (31517) | 93.9 (10065) | 87.1 (21452) |  |
| On dialysis at transplant^d^ |  |  |  | <0.001 |
| Yes | 9.5 (3355) | 5.2 (556) | 11.4 (2797) |  |
| No | 90.5 (31985) | 94.8 (10161) | 88.6 (21824) |  |
| MELD score at transplant, median (IQR) | 19 (13-29) | 16 (11-24) | 21 (14-31) | <0.001 |
| **Transplant Characteristics** |  |  |  |  |
| Donor type |  |  |  | <0.001 |
| Living donor | 4.3 (1532) | 6.6 (706) | 3.4 (826) |  |
| Deceased donor | 95.7 (33808) | 93.4 (10012) | 96.6 (23796) |  |
| Time period |  |  |  | <0.001 |
| 2010-2014 | 51.5 (18198) | 49.0 (5247) | 52.6 (12951) |  |
| 2015-2018 | 48.5 (17142) | 51.0 (5471) | 47.4 (11671) |  |

Abbreviations: BMI, body mass index; HCC, hepatocellular carcinoma; ICU, intensive care unit; IQR, interquartile range; MELD, Model for End-Stage Liver Disease score; NASH, non-alcoholic steatohepatitis; OR, odds ratio.

^a^Missing n=1569; ^b^Missing n=86; ^c^Missing n=262; ^d^Missing n=2

Supplemental Table 2. Factors Associated with New Employment Post-Transplant.

|  | **Employed Post-Transplant,** n=4489, % (n) | **Not Employed Post-Transplant,** n=20133, % (n) | **p-value** | **Employed Post-Transplant,** UV OR (95% CI) | **p-value** | **Employed Post-Transplant,** MV OR (95% CI) |
| --- | --- | --- | --- | --- | --- | --- |
| Age |  |  | <0.001 |  | <0.001 |  |
| 18-39 | 32.1 (679) | 67.9 (1439) |  | Ref |  | Ref |
| 40-49 | 27.9 (1050) | 72.1 (2710) |  | 0.82 (0.73-0.92) |  | 0.86 (0.76-0.98) |
| 50-59 | 18.0 (1945) | 82.0 (8878) |  | 0.46 (0.42-0.52) |  | 0.50 (0.45-0.57) |
| ≥60 | 10.3 (815) | 89.7 (7106) |  | 0.24 (0.22-0.27) |  | 0.26 (0.23-0.30) |
| Sex |  |  | <0.001 |  | <0.001 |  |
| Male | 20.5 (3273) | 79.5 (12696) |  | 1.58 (1.47-1.69) |  | ^e^ |
| Female | 14.0 (1216) | 86.0 (7437) |  | Reference |  | Reference |
| BMI |  |  | 0.02 |  | 0.02 |  |
| Underweight | 16.4 (82) | 83.6 (419) |  | 0.81 (0.63-1.03) |  |  |
| Normal | 19.5 (1232) | 80.5 (5096) |  | Reference |  |  |
| Overweight | 18.1 (1501) | 81.9 (6773) |  | 0.92 (0.84-1.00) |  |  |
| Obese | 17.6 (1674) | 82.4 (7845) |  | 0.88 (0.81-0.96) |  |  |
| Ethnicity/race |  |  | <0.001 |  | <0.001 | ^e^ |
| Non-Hispanic White | 19.1 (3294) | 80.9 (13974) |  | Ref |  |  |
| Non-Hispanic Black | 18.0 (383) | 82.0 (1740) |  | 0.93 (0.83-1.05) |  |  |
| Hispanic | 14.4 (569) | 85.6 (3379) |  | 0.72 (0.65-0.79) |  |  |
| Other | 18.9 (243) | 81.1 (1040) |  | 0.99 (0.86-1.15) |  |  |
| Educational attainment^a^ |  |  | <0.001 |  | <0.001 |  |
| Less than high school | 10.5 (158) | 89.5 (1352) |  | 0.64 (0.54-0.77) |  | 0.89 (0.74-1.07) |
| High school or equivalent | 15.3 (1719) | 84.7 (9488) |  | Reference |  | Reference |
| College | 21.4 (2062) | 78.6 (7570) |  | 1.50 (1.40-1.61) |  | 1.35 (1.25-1.46) |
| Post-graduate | 30.0 (318) | 70.0 (743) |  | 2.36 (2.05-2.72) |  | 2.07 (1.78-2.42) |
| Payment source |  |  | <0.001 |  | <0.001 |  |
| Private insurance | 26.0 (3083) | 74.0 (8792) |  | Reference |  | Reference |
| Public insurance | 10.9 (1364) | 89.1 (11147) |  | 0.35 (0.32-0.37) |  | 0.36 (0.34-0.39) |
| Other | 17.8 (42) | 82.2 (194) |  | 0.62 (0.44-0.86) |  | 0.46 (0.32-0.67) |
| **Medical Condition at Transplant** |  |  |  |  |  |  |
| Diabetes^b^ |  |  | <0.001 |  | <0.001 |  |
| Yes | 13.3 (853) | 86.7 (5576) |  | 0.61 (0.56-0.66) |  | 0.75 (0.69-0.82) |
| No | 20.0 (3622) | 80.0 (14503) |  | Reference |  | Reference |
| Prior ascites |  |  |  |  |  |  |
| Yes | 18.7 (3616) | 81.3 (15758) | 0.001 | 1.15 (1.06-1.25) | 0.001 |  |
| No | 16.6 (873) | 83.4 (4375) |  | Reference |  |  |
| Prior encephalopathy |  |  | 0.66 |  | 0.66 |  |
| Yes | 18.3 (3026) | 81.7 (13502) |  | 1.02 (0.95-1.09) |  |  |
| No | 18.1 (1463) | 81.9 (6631) |  | Reference |  |  |
| Impaired functional status^c^ |  |  |  |  | 0.03 |  |
| Yes | 18.4 (3983) | 81.6 (17613) | 0.03 | 1.12 (1.01-1.24) |  |  |
| No | 16.8 (482) | 83.2 (2391) |  | Reference |  |  |
| Primary etiology of liver disease |  |  | <0.001 |  | <0.001 |  |
| Viral | 15.4 (983) | 84.6 (5419) |  | Reference |  | Reference |
| Alcohol-related | 21.8 (1464) | 78.2 (5241) |  | 1.54 (1.41-1.68) |  | 1.01 (0.92-1.12) |
| NASH | 15.2 (437) | 84.8 (2448) |  | 0.98 (0.87-1.11) |  | 0.98 (0.85-1.12) |
| Autoimmune/cholestatic | 23.1 (532) | 76.9 (1768) |  | 1.66 (1.47-1.87) |  | 1.21 (1.05-1.39) |
| HCC | 12.8 (510) | 87.2 (3485) |  | 0.81 (0.72-0.90) |  | 1.02 (0.90-1.15) |
| Other | 24.1 (563) | 75.9 (1772) |  | 1.75 (1.56-1.97) |  | 1.28 (1.12-1.46) |
| On ventilator at transplant |  |  | 0.51 |  | 0.51 |  |
| Yes | 17.4 (163) | 82.6 (773) |  | 0.94 (0.79-1.12) |  |  |
| No | 18.3 (4326) | 81.7 (19360) |  | Reference |  |  |
| In ICU at transplant |  |  | <0.001 |  | <0.001 |  |
| Yes | 21.6 (683) | 78.4 (2487) |  | 1.27 (1.16-1.40) |  |  |
| No | 17.7 (3806) | 82.3 (17646) |  | Reference |  |  |
| On dialysis at transplant^d^ |  |  | <0.001 |  | <0.001 |  |
| Yes | 21.6 (604) | 78.4 (2193) |  | 1.27 (1.16-1.40) |  |  |
| No | 17.8 (3885) | 82.2 (17939) |  | Reference |  |  |
| MELD score at transplant median (IQR) | 24 (16-34) | 20 (14-30) | 0.001 | 1.03 (1.02-1.03) | 0.001 | 1.02 (1.01-1.02) |
| **Transplant Characteristics** |  |  |  |  |  |  |
| Donor type |  |  | 0.02 |  | 0.02 |  |
| Living donor | 21.2 (175) | 78.8 (651) |  | 1.21 (1.02-1.44) |  |  |
| Deceased donor | 18.1 (4314) | 81.9 (19482) |  | Reference |  |  |
| Time period |  |  | 0.18 |  | 0.18 |  |
| 2010-2014 | 17.9 (2321) | 82.1 (10630) |  | Reference |  | Reference |
| 2015-2018 | 18.6 (2168) | 81.4 (9503) |  | 1.04 (0.98-1.12) |  | 1.13 (1.05-1.22) |

Shown is a comparison of the characteristics of transplant recipients not employed pre-transplant by post-transplant employment status and a logistic regression model evaluating the factors associated with post-transplant employment in this population.

Abbreviations: BMI, body mass index; HCC, hepatocellular carcinoma; ICU, intensive care unit; IQR, interquartile range; MELD, Model for End-Stage Liver Disease score; NASH, non-alcoholic steatohepatitis; OR, odds ratio.

^a^Missing n=1212; ^b^Missing n=68; ^c^Missing n=153; ^d^Missing n=1; ^e^Significant interaction between race and sex. Female: non-Hispanic Black vs. non-Hispanic White: OR 1.36 (95% CI 1.11-1.67), Hispanic vs. non-Hispanic White: OR 0.77 (95% CI 0.62-0.95), other vs. non-Hispanic White: OR 0.86 (95% CI 0.63-1.19). Male: non-Hispanic Black vs. non-Hispanic White: OR 0.91 (95% CI 0.77-1.08), Hispanic vs. non-Hispanic White: OR 0.91 (95% CI 0.77-1.08), other vs. non-Hispanic White: OR 1.20 (95% CI0.99-1.44)
